# Incidence, seasonal pattern, and clinical manifestations of S*treptococcus dysgalactiae* subspecies *equisimilis* bacteremia; a population-based study

**DOI:** 10.1007/s10096-023-04607-8

**Published:** 2023-04-29

**Authors:** Viivi Nevanlinna, Reetta Huttunen, Janne Aittoniemi, Tiina Luukkaala, Sari Rantala

**Affiliations:** 1 Department of Internal Medicine, Tampere University Hospital, Elämänaukio, Kuntokatu 2, 33520 Tampere, Finland; 2 Faculty of Medicine and Health Technology, Tampere University, Tampere, Finland; 3 Fimlab Laboratories, Tampere, Finland; 4 Research, Development and Innovation Center, Tampere University Hospital, Tampere, Finland; 5 Health Sciences, Faculty of Social Sciences, Tampere University, Tampere, Finland

**Keywords:** *Streptococcus dysgalactiae* subspecies *equisimilis*, Group G Streptococci, Group C Streptococci, Bacteremia, Incidence, Seasonality

## Abstract

*Streptococcus dysgalactiae* subspecies *equisimilis* (SDSE) is a human pathogen causing severe invasive infections. Population-based studies on SDSE bacteremia are limited. The purpose of this study was to investigate the incidence, seasonal pattern, clinical manifestations, and recurrence of SDSE bacteraemia. Records regarding patients aged ≥ 18 years with SDSE bacteremia in the Pirkanmaa health district in August 2015 to July 2018 were retrospectively reviewed. A total of 230 SDSE bacteremia episodes were identified, with 217 episodes (involving 211 patients) available for analysis. The mean annual incidence rate of SDSE bacteremia was 16.9/100 000 inhabitants. Most episodes (33%) were detected in the summer (June to August) (*p* = 0.058). Episodes with bacteremic cellulitis were statistically significantly more common during the summer compared with other seasons (*p* = 0.008). Cellulitis was the most common presenting clinical manifestation of SDSE bacteremia (68% of all episodes). Risk factors of recurring bacteremia were chronic eczema and/or skin erosion (OR 3.96 [95% CI 1.11–14.1]), heart disease (OR 3.56 [95% CI 1.22–10.4]), diabetes (OR 3.77 [95% CI 1.35–10.5]) and a history of cellulitis. We found a remarkably high incidence of SDSE bacteraemia in the Pirkanmaa health district. Bacteraemic cellulitis, which was the predominant clinical manifestation is more often occurred in the summer. Risk factors of recurring SDSE bacteremia were a history of cellulitis, chronic eczema or skin erosion, diabetes, and heart disease.

## Introduction

*Streptococcus dysgalactiae* subspecies *equisimilis* (SDSE) is a human pathogen including almost all ß-haemolytic large colony-forming group C and G streptococci [1]. Several investigators have reported an increase in the incidence of SDSE bacteremia both in Finland and other countries [2–4]. In the National Infectious Disease Register in Finland, SDSE was the fourth most common pathogen causing bacteremia, and has been three-times more common than *Streptococcus pyogenes* in the past few years [5]. Detailed information on the epidemiology and clinical manifestations of SDSE bacteremia is limited.

In bacteremia, seasonality has been previously reported in streptococcal and pneumococcal diseases. In an invasive pneumococcal disease, the incidence is typically higher in late winter and early spring, and lower in summer and autumn [6]. *Streptococcus pyogenes* bacteremia shows similar seasonal variation [7]. The seasonal pattern of SDSE bacteremia has been previously investigated in only a few studies [3, 8, 9]. In two studies, SDSE bacteremia was found to occur more frequently in the warm season [3, 8].

SDSE is part of the normal flora of the pharynx, skin, gastrointestinal tract, and female genital tract, but it also causes severe invasive infections [10]. The most common clinical manifestation of SDSE bacteremia is cellulitis, which has been shown to occur in 43 to 57 per cent of SDSE bacteremia episodes [2, 4, 11]. Other clinical manifestations of SDSE bacteremia are pneumonia, septic arthritis, osteomyelitis, deep abscesses, endocarditis, necrotizing fasciitis, and streptococcal toxic shock syndrome [2, 4, 11]. In 16–28% of episodes, no defined focus is found [2, 4, 11].

In previous studies, SDSE bacteremia has been reported to recur in 3–8% of patients [12–15]. In the great majority of patients with recurrent bacteremia, the site of infection has been the skin, presenting as cellulitis [12–15]. A history of cellulitis and lymphatic abnormalities has been suggested to be a risk factor of recurrent SDSE bacteremia [14, 15]. Although a significant proportion of cases of SDSE bacteremia tend to recur, only a few studies have been carried out to investigate the phenomenon [12–17].

The incidence of SDSE bacteremia seems to be increasing, but population-based studies, in particular, are limited and updated information on the epidemiology of SDSE bacteremia is needed.

The objective of this study was to investigate the incidence, seasonal pattern, clinical manifestations, and recurrence of SDSE bacteremia.

## Methods

The Pirkanmaa health district is the second-largest health district in Finland, with 535 044 inhabitants. It comprises one tertiary care hospital (Tampere University hospital), four regional hospitals and several smaller healthcare units. In the Pirkanmaa health district, all positive blood-culture results are registered into a database (SAI), maintained by the Department of Hospital Hygiene and Infection Control. All adult (≥ 18 years old) SDSE bacteremia episodes treated in the hospitals of the Pirkanmaa health district from June 2015 to August 2018 were retrospectively identified in the SAI register. Patients with at least one positive blood culture were included in the study. All patients also had clinical signs compatible with infection. During the study period, 230 blood cultures positive for SDSE were identified, and medical records were available for 217 episodes, involving 211 patients. An infectious-disease specialist (SR) reviewed the electronic patient records and filled in a structured case report form. Patient characteristics of the study population have been described in detail elsewhere [18]. The study was approved by the Regional Ethics Committee of Tampere University Hospital.

All blood-culture samples in the Pirkanmaa area were studied and cultivated in the Fimlab laboratories, Tampere. Blood cultures collected from August 2015 to October 2017 were collected into BacT/Alert Aerobic (FA Plus) and Anaerobic (FN Plus) blood-culture bottles and incubated in an automated microbial detection system (BacT/Alert 3D, bioMérieux, Marcy l’Etoile, France). From November 2017 to July 2018 the blood cultures were collected into BD BACTEC Plus Aerobic/F and Lytic/10 Anaerobic/F culture vials and incubated in a BD BACTEC FX blood-culture system (Becton Dickinson, Sparks, MD, USA). SDSE was primarily determined on the basis of typical large colony-forming growth and β-haemolysis on blood agar plates. Up until February 2017, identification of the bacteria was primarily based on latex agglutination in Lancefield grouping (PathoDxtraTM Strep Grouping Kit, Thermo Scientific, Basingstoke, Hants, UK), and confirmation on API® 20 STREP (bioMérieux, Marcy l’Etoile, France) or matrix-assisted laser-desorption/ionization time-of-flight mass spectrometry, i.e., MALDI-TOF MS (VITEK® MS, bioMérieux, Marcy l’Etoile, France) [19]. Since March 2017, the MALDI-TOF method has been used for identification. MALDI-TOF gives the result in the form of *S. dysgalactiae* subsp. *dysgalactiae/equisimilis*, interpreted as *S. dysgalactiae* subsp. *equisimilis* associated with human disease.

To calculate the age- and sex-specific incidence rates of SDSE bacteremia, we used data provided by Statistics Finland regarding the population in the Pirkanmaa health district (https://stat.fi/index_en.html). To calculate the overall and age-specific incidences, the number of SDSE bacteremia patients under 18 years of age during the study period was obtained from the Finnish National Infectious Disease Register [5].

SDSE bacteremia was defined as recurrent if a prior SDSE episode had occurred at least three weeks earlier and the first episode had been treated adequately. Associations between the risk factors and recurrent SDSE bacteraemia were assessed by using the χ^2^ test or Fisher’s exact test, as appropriate. Univariable logistic regression analyses were performed to investigate possible risk factors of recurrent SDSE bacteremia. Owing to quasi-complete separation, it was not possible to include previous cellulitis as a risk factor in the logistic regression analyses. Odds ratios were calculated, with 95% confidence intervals. P-values under 0.05 were considered statistically significant. Statistical analyses were performed with IBM SPSS Statistics for Mac, Version 27 software (IBM Corp, Armonk, NY, US).

## Results

**Fig. 1:**
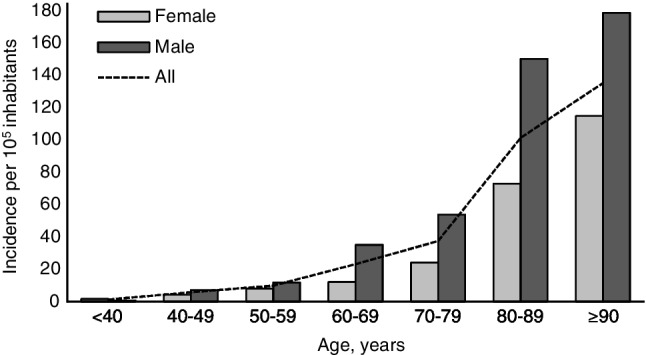
Age-related mean annual incidence rates of *Streptococcus dysgalactiae* subs. *equisimilis* bacteremia in the Pirkanmaa health district during the study period of August 2015 to July 2018

The study concerned 217 episodes of SDSE bacteremia, involving 211 patients. The median age was 75 years (range 28 to 95 years) and 60% of the patients were male. The mean annual incidence rate of SDSE bacteremia in all patients was 16.9/100 000 inhabitants, range 14.8 to 18.9/100 000 inhabitants. There was no statistically significant trend in incidence rates during the three-year study period. The age-related mean annual incidence rates are shown in Fig. 1. Incidence rates were higher in male (20.8/100 000) than in female patients (13.3/100 000) in the whole study group and in all age groups, but statistical significance was not reached (*p* = 0.317). In patients over 60 years old, the incidence rate was approximately twofold in males compared with females. The incidence was highest in the oldest age group (≥ 90 years of age; 135.0/100 000).

**Fig. 2:**
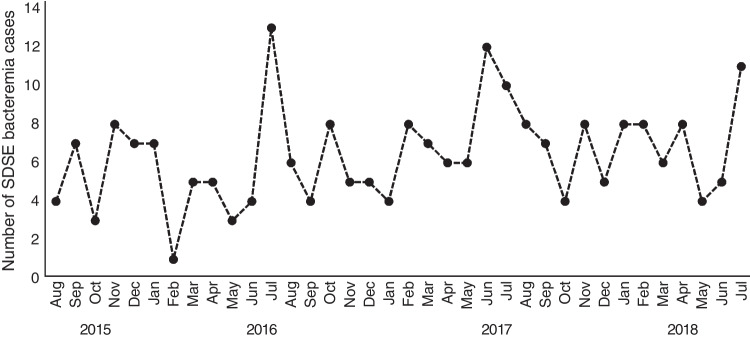
Seasonality *of Streptococcus dysgalactiae* subs. *equisimilis* bacteremia in the Pirkanmaa health district from August 2015 to July 2018

**Fig. 3:**
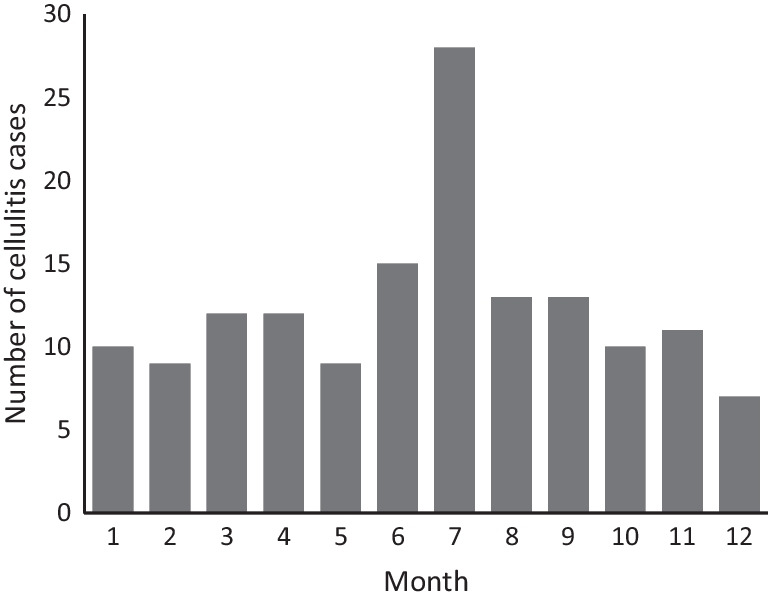
Seasonality of cellulitis as presenting clinical manifestation of *Streptococcus dysgalactiae* subsp. *equisimili*s bacteremia during the study period of August 2015 to July 2018

The seasonality of SDSE bacteremia is shown in Fig. 2. SDSE bacteremia episodes were most often detected during the summer months (June to August; 33% of all episodes). The fewest episodes (20%) were detected in spring (March to May). A total of 24% of the episodes occurred in autumn (September to November), and 24% in winter (December to February). However, seasonal variation of all SDSE bacteremia episodes did not reach statistical significance (*p* = 0.058). The seasonal pattern was most prominent in episodes with cellulitis as the presenting clinical manifestation (*p* = 0.008), as shown in Fig. 3. Thirty-eight per cent of cellulitis episodes leading to SDSE bacteremia occurred during the summer, and the highest peak in incidence was seen in June (19%). The fewest episodes were detected during winter (17%). Twenty-two per cent of cellulitis episodes were detected in spring and 23% in autumn.

The clinical manifestations of SDSE bacteremia are presented in Table 1. Seventy-three per cent of the patients had a skin and soft tissue infection, and cellulitis was the most common presenting clinical manifestation (68%). Other clinical manifestations were pneumonia (16%), deep abscess (7%) and bone and joint infections (4%). In thirteen per cent of SDSE episodes, the site of the infection was not determined. We present the most common clinical manifestations according to age groups in Table 2. There was no distinct difference in the proportion of cases of cellulitis or other skin and soft tissue infections between the different age groups. There appeared to be a trend for pneumonia to be more common in older age groups, but no statistical significance was found. The site of infection appeared to be more often known in younger patients, but the results did not reach statistical significance. When the patients were analysed in two different age groups, i.e., under 80 years and ≥ 80 years of age, pneumonia was a statistically significantly more frequent clinical manifestation in the older patients (*p* = 0.039). The 30-day case fatality rate after the first positive blood culture was 8% (17/217 patients).

**Table 1:** Clinical manifestations of *Streptococcus dysgalactiae* subsp. *equisimilis (*SDSE) bacteremia during the study period of August 2015 to July 2018

Site of infection^a^	Episodes of SDSE bacteremia, *N* = 217	
	n	(%)
Skin/soft tissue infection	158	(73)
Cellulitis	149	(68)
Purulent skin infection	43	(20)
Necrotizing fasciitis	1	(0.5)
Deep abscess	14	(7)
Bursitis	3	(1)
Bone and joint infection	9	(4)
Osteomyelitis	3	(1)
Spondylitis	2	(1)
Arthritis	8	(4)
Periprosthetic joint infection	4	(2)
Pneumonia	35	(16)
Empyema	2	(1)
Endocarditis	3	(1)
Aortitis	1	(0.5)
Foreign body infection	3	(1)
Urinary tract infection	3	(1)
Puerperal sepsis	4	(2)
Intra-abdominal infection	3	(1)
Endophthalmitis	1	(0.5)
Bacteremia without a defined focus	29	(13)

^a^One patient may have one or more clinical manifestations

**Table 2:** The most common clinical manifestations of *Streptococcus dysgalactiae* subsp. *equisimilis* bacteremia by age during the study period of August 2015 to July 2018

Site of infection^a^	18–49 years *N* = 17 (%)		50–64 years *N* = 33 (%)		65–79 years *N* = 83 (%)		≥ 80 years *N* = 84 (%)		*P*-value
Skin/soft tissue infection	14	(82)	24	(73)	66	(80)	57	(68)	0.306
Cellulitis	13	(76)	24	(73)	60	(72)	52	(62)	0.388
Purulent skin infection	5	(29)	8	(23)	17	(20)	13	(15)	0.492
Pneumonia	0	(0)	4	(12)	12	(14)	19	(23)	0.092
Deep abscess	3	(18)	3	(9)	3	(4)	5	(6)	0.114
Bone and joint infection	1	(6)	2	(6)	3	(4)	3	(4)	0.724
Arthritis	1	(6)	1	(3)	3	(4)	3	(4)	0.891
Bacteremia without defined focus	1	(6)	3	(9)	10	(12)	15	(18)	0.478

^a^One patient may have one or more clinical manifestations

A total of 18 patients (9%) had recurrent SDSE bacteremia. The median time from the previous episode of SDSE bacteremia was 15 months (range 1 to 110 months). In patients suffering from SDSE bacteraemia for the first time, only 32% had a history of previous cellulitis, whereas all patients with recurring bacteremia had a history of one or more episodes of cellulitis. Of those suffering from bacteremia for the first time, 67% had cellulitis as the presenting clinical manifestation. In recurring SDSE episodes, 85% had cellulitis as the presenting clinical manifestation. Risk factors of recurrent SDSE bacteremia were studied. Chronic eczema or skin erosion increased the risk of recurrent bacteremia (univariable Odds Ratio, OR, 3.96, [95% Confidence Interval, CI, 1.11–14.1]). Diabetes (OR 3.77 [95% CI 1.35–10.5]) and heart disease (OR 3.56 [95% CI 1.22–10.4]) were also associated with an increased risk of recurrent SDSE bacteremia. Owing to quasi-complete separation, it was not possible to calculate the odds ratio of previous cellulitis being a risk factor of recurrent SDSE bacteremia. Alcoholism, smoking, obesity, pulmonary disease, immunosuppression, and malignancy were also studied as risk factors, but no association with recurrence of SDSE bacteremia was found. Age and sex were not found to increase the risk of recurrent bacteremia.

## Discussion

The results of the present study revealed a remarkably higher incidence of SDSE bacteremia compared with previously reported incidences in the same population [2, 20]. In the Pirkanmaa health district, the incidence of SDSE bacteremia has been previously reported to have increased from 2.05 to 4.75 episodes per 100 000 inhabitants from 1995 to 2004, and to 6.2/100 000 in 2009 [2, 20]. In our study, the mean annual incidence was found to have increased to 16.9/100 000 inhabitants.

The incidence of SDSE bacteremia has been reported to have increased in other countries as well. In a recent study from Norway, the incidence of SDSE bacteremia was reported to have increased from 1.4 to 7.6/100 000 inhabitants during 1999–2021 and it is now the leading cause of β-haemolytic streptococcal bloodstream infections in that country [21]. In Denmark, the highest incidence reported was 3.5/100 000 inhabitants in 2009 for Group G streptococci [22].

In our study, the incidence was higher in male patients and it increased dramatically in patients over 60 years of age in both sexes. The distributions of sex- and age-related incidences in a study by Oppegaard et al. were similar to those in the present study, but the incidence rates were much lower in all age groups [3]. Kontula et al. studied all bloodstream infections in Finland from 2004 to 2018 and found that the overall incidence had risen from 150 to 309 episodes per 100 000 inhabitants, and that the increase in incidence was highest amongst the oldest persons [23]. In Norway, the incidence of all bloodstream infections rose from 190 to 257 episodes per 100 000 inhabitants during 2002–2013 [24].

There are several reasons for the increase in incidence of SDSE bacteremia. The majority of SDSE bacteremia patients are older people with several comorbidities, and the patients are more often male than female. Both in our study and in other studies concerning bloodstream infections, the increase in incidence is most notable in persons over 60 years of age. Ageing is known to increase the risk of infection in many types of infectious disease as a result of the higher prevalence of comorbidities, and also because of alterations in the immune system [25]. In developed countries, the population is ageing, and life expectancy has risen [26]. The treatment of chronic diseases has improved, and the prevalence of chronic diseases is higher than earlier [27].

The incidence of all bloodstream infections in Finland and other countries is increasing, but the increase in the incidence of SDSE bacteremia seems to be especially high. According to the National Infectious Disease Register in Finland, SDSE has been the fourth most common causative agent in bacteremia, and three times more common compared with *S. pyogenes* in recent years [5]. The incidence of *S. pyogenes* has been reported to have increased as well, but compared with SDSE, the incidence rates are low. In Southwest Finland, the incidence of *S. pyogenes* bacteremia has been reported to have increased from 2.59 to 5.23/100 000 persons in the period of 2007–2018 [28]. Indications of SDSE surpassing *S. pyogenes* in bacteremic infections have been reported in Norway, Denmark and Japan as well [3, 22, 29].

Virulence factors shared between *S. pyogenes* and SDSE are well known. Recently, evidence of horizontal gene transfer between SDSE and *S. pyogenes* has been presented, with a preferred gene flow from *S. pyogenes* to SDSE [30]. Genomic analyses of isolates from invasive SDSE infections have shown that a limited number of clonal groups are responsible for the increasing amount of SDSE bacteremia episodes [29, 31, 32]. These findings suggest that a few highly virulent SDSE clones might be responsible for the increasing incidence of SDSE bacteremia [29, 31, 32]. In Norway, a virulent genotype, StG62647, rarely seen before 2012, has emerged as the cause of 20% of all SDSE bacteremia episodes, comprising ﻿80% of invasive group C streptococcal infections in 2015 [31]. In Sweden and Austria, the same genotype has been associated with an invasive phenotype and more severe clinical manifestations [33, 34].

In our study, bacteremia with cellulitis as a clinical manifestation was statistically significantly more common during summer compared with other seasons. To our knowledge, this is the first study carried out to address the seasonality of SDSE in bacteremic cellulitis. In previous studies, warm weather has been shown to increase the risk of cellulitis. Peterson et al. demonstrated a dose-dependent relationship between temperature and the odds of hospital admission for cellulitis [35]. Other risk factors associated with cellulitis are skin trauma, venous insufficiency or lymphoedema, older age, and obesity [36–38].

Of all episodes of bacteremia, 33% occurred during the summer, but the seasonal variation did not reach statistical significance. The seasonal pattern of SDSE bacteremia has been previously examined in only a few studies [3, 8, 9]. Oppegaard et al. presented similar results; 31% of cases of SDSE bacteremia occurred during the summer months [3]. In a single-centre study with 52 patients in Korea, 65.4% of the episodes occurred during the warm season [8].

Our study showed that a skin infection is the dominant source of SDSE bacteremia. It seems that the proportion of cases of cellulitis as a presenting clinical manifestation has increased in SDSE bacteremia. In our study, 68% of the patients had cellulitis as a presenting clinical manifestation, and in previous studies the proportion has varied from 34 to 57 per cent [2, 4, 11]. *Streptococcus pyogenes* has traditionally been thought to be the main causative pathogen in cellulitis. However, in a previous Finnish study, SDSE, instead of *S. pyogenes*, was found to predominate in bacterial cellulitis [39]. It seems that compared with *S. pyogenes*, SDSE is the causative agent of cellulitis in a larger proportion of cases than earlier. These results might partly explain the increasing incidence of SDSE bacteraemia.

The second most common presenting clinical manifestation was pneumonia, in 16% of the episodes, and it was more common in elderly patients. In previous studies, pneumonia has been reported in only 5–9% of episodes [2, 4, 11]. More serious clinical manifestations, including endocarditis, osteomyelitis, spondylitis, and necrotizing fasciitis were relatively rare in our study population. Bacteremia without a defined focus has been previously reported in 16–28% of cases [2, 4, 11]. In our study, only 13% of the cases did not have a defined focus. In elderly patients, the site of infection seemed to be more often unknown. There are only a few previous studies on the relationship between age and clinical manifestations in SDSE bacteremia, but the results are in line with ours [29, 40]. Pneumonia has been reported to occur more often in older patients [29]. Abscesses have been more common focuses in young patients and the site of infection seems to be more often known in younger patients [29]. In our study, the case fatality rate was slightly lower compared with those reported in previous population-based studies (15–20%) [3, 4, 11, 12].

Previous studies on the risk factors of recurring SDSE bacteremia are limited [12–17]. Consistent with our results, a history of cellulitis has been previously associated with recurring SDSE bacteremia [13, 14]. Recurring SDSE bacteremia seems to be strongly associated with a history of cellulitis, and in the great majority of recurring SDSE bacteremia episodes, cellulitis also presents as the clinical manifestation. In previous studies, chronic lymphatic abnormalities and genital cancer have been identified as other risk factors [14, 15]. We also found chronic eczema or skin erosion to be associated with recurrence. Furthermore, we present the first study with chronic diseases including diabetes and heart diseases as risk factors of recurring SDSE bacteremia. Patients with SDSE bacteremia are typically elderly individuals with chronic diseases, and males are affected more often than females. However, when focusing on recurring bacteremia, age and sex do not seem to be associated with the risk of recurrence.

Our study has several strengths. The study population is relatively large compared with those in several other studies on SDSE bacteremia. The medical records of the patients were comprehensively available (217/230) and same infectious-disease specialist (SR) reviewed all of the records. Our study is population-based and might give a more realistic spectrum of the clinical manifestations in comparison with several previous single-centre studies.

There are also some limitations. The study is retrospective by its nature. The increase in the incidence of SDSE bacteremia is remarkable, when compared with rates reported in previous studies. However, in our study, the three-year study period was too short to demonstrate the increase in incidence during that period. In the future, more studies and updated information on the incidence of SDSE bacteraemia are needed.

## Conclusion

In conclusion, the present study showed a remarkably high incidence of SDSE bacteremia in the Pirkanmaa health district, Finland. The incidence has increased dramatically in comparison with rates reported in previous studies in the same area. SDSE bacteremia predominantly presents with cellulitis as its clinical manifestation. A novel finding was that bacteremic cellulitis occurs more often during the warm season. Risk factors of recurring SDSE bacteremia are a history of cellulitis, chronic eczema or skin erosion, diabetes, and heart disease. These results emphasize the importance of SDSE as a causative agent of bacteremia. More studies on the epidemiology of SDSE bacteremia, including recent incidence rates, are needed.

## Data Availability

The dataset is not publicly available owing to individual privacy, but it is available from the corresponding author on reasonable request.
